# Liraglutide modulates GABAergic signaling in rat hippocampal CA3 pyramidal neurons predominantly by presynaptic mechanism

**DOI:** 10.1186/s40360-017-0191-0

**Published:** 2017-12-16

**Authors:** Omar Babateen, Sergiy V. Korol, Zhe Jin, Amol K. Bhandage, Aikeremu Ahemaiti, Bryndis Birnir

**Affiliations:** Department of Neuroscience, Uppsala University, 75124 Uppsala, SE Sweden

**Keywords:** GABA, GLP-1 receptor, Patch-clamp, Inhibitory postsynaptic and tonic currents, Hippocampus, Electrophysiology

## Abstract

**Background:**

γ-Aminobutyric acid (GABA) is the main inhibitory neurotransmitter in the brain where it regulates activity of neuronal networks. The receptor for glucagon-like peptide-1 (GLP-1) is expressed in the hippocampus, which is the center for memory and learning. In this study we examined effects of liraglutide, a GLP-1 analog, on GABA signaling in CA3 hippocampal pyramidal neurons.

**Methods:**

We used patch-clamp electrophysiology to record synaptic and tonic GABA-activated currents in CA3 pyramidal neurons in rat hippocampal brain slices.

**Results:**

We examined the effects of liraglutide on the neurons at concentrations ranging from one nM to one μM. Significant changes of the spontaneous inhibitory postsynaptic currents (sIPSCs) were only recorded with 100 nM liraglutide and then in just ≈50% of the neurons tested at this concentration. In neurons affected by liraglutide both the sIPSC frequency and the most probable amplitudes increased. When the action potential firing was inhibited by tetrodotoxin (TTX) the frequency and amplitude of IPSCs in TTX and in TTX plus 100 nM liraglutide were similar.

**Conclusions:**

The results demonstrate that liraglutide regulation of GABA signaling of CA3 pyramidal neurons is predominantly presynaptic and more limited than has been observed for GLP-1 and exendin-4 in hippocampal neurons.

## Background

The hippocampus is a brain structure well recognized for its involvement in cognitive functions [1, 2]. What is not as widely known is that the hippocampus may influence metabolic functions. Hypothalamic neurons are inhibited in a topographical manner by outputs from the hippocampus [3, 4] and the hippocampus expresses receptors for a number of metabolic hormones including insulin and GLP-1 [5, 6]. Thus feedback regulation of hippocampal functions by molecules of the metabolic system can take place [3].

Diabetes mellitus type-2 is a progressive disease associated with insulin resistance with resulting high blood glucose concentration and, with time, suboptimal functioning of a number of organs. In the brain, type-2 diabetes has been shown to increase the risk for dementia and Alzheimer disease [7–9]. Pilot studies using insulin have been promising in patients with cognitive impairments and have demonstrated significant improvements in memory formation [10]. The GLP-1, and mimetics like exendin-4 (exenatide) and liraglutide, activate GLP-1 receptor signaling pathways that converge with the insulin-activated intracellular signaling pathway [11]. The GLP-1 receptor is widely distributed in the brain and its activation has been shown to be neuroprotective, anti-inflammatory, to regulate food intake, synaptic plasticity and memory formation [11–13]. The metabolic hormones clearly have vital functions in the healthy as well as the diseased brain.

γ-Aminobutyric acid (GABA), is the main inhibitory neurotransmitter in the central nervous system (CNS) and activates GABA-A receptors that are chloride permeable ion channels, and the GABA-B receptor, a G-protein coupled receptor [14]. The GABA-A receptors mediate synaptic and tonic inhibitory currents and thereby regulate excitability of neurons and neuronal networks [15]. In 1984, Palovick et al. demonstrated that insulin inhibits hippocampal pyramidal neurons [16] and in recent years, it has become apparent that metabolic hormones enhance GABA signaling in hippocampal neurons. Insulin increases miniature inhibitory postsynaptic currents (mIPSCs) in cultured hippocampal neurons [17] and turns-on high-affinity GABA-A receptors generating tonic currents in rat hippocampal CA1 pyramidal neurons [18] decreasing neuronal excitability. We recently have shown in rat hippocampal CA3 neurons that physiological concentrations of GLP-1 (10 pM) and clinically relevant concentrations of exendin-4 transiently enhance the synaptic and tonic currents [19, 20].

In the current study we have examined what effects liraglutide, a long-acting GLP-1 receptor agonist, might have on the GABA signaling in the rat hippocampal CA3 pyramidal neurons. Liraglutide has 97% amino acid homology with the native human GLP-1 protein. It differs from GLP-1-(7–37) by one amino acid, an arginine 34 that is substituted for a lysine, and a 16-carbon fatty acid side chain that is attached at the level of lysine 26 [21]. The results presented here reveal that liraglutide partially mimics the effects of GLP-1 and exendin-4 in rat CA3 pyramidal neurons.

## Methods

### Hippocampal slice preparation

Hippocampal slices from 16 to 22 days old Wistar rats (Taconic Biosciences (Denmark) and Charles River (Germany)) were used for electrophysiological recordings. All experimental animal procedures were conducted in line with the local ethical guidelines and protocols approved by the Uppsala Animal Ethical Board (Uppsala, Sweden), Dnr C192/14. Hippocampal slices were prepared as previously described [19]. Briefly, the animal was decapitated, the brain was removed and immersed into an ice-cold artificial cerebrospinal fluid (ACSF). It contained (in mM): 124 NaCl, 3 KCl, 2.5 CaCl_2_, 1.3 MgSO_4_, 26 NaHCO_3_, 2.5 Na_2_HPO_4_, and 10 glucose, pH 7.3–7.4 when the solution was bubbled with 95% O_2_ and 5% CO_2_. Hippocampal slices, 400 μm thick, were prepared in ice-cold ACSF. The slices were then incubated for one hour at 37 °C and then kept at room temperature (20-22 °C) until used in experiments.

### Electrophysiological recording and analysis

All electrophysiological recordings and analysis were as previously described [19]. The composition of the pipette solution in mM was: 140 CsCl, 1 CaCl_2_, 3 EGTA, 0.5 KCl, 1 MgCl_2_, 2 ATP-Mg, 0.3 GTP-Na, 5 QX-314 bromide, and 10 TES (pH 7.25 adjusted with CsOH). The holding potential was −60 mV in all experiments. The ACSF with kynurenic acid, an antagonist at excitatory glutamate receptors (3 mM), plus the drugs were perfused during experiments. Drugs were in general purchased from Sigma-Aldrich (Germany). Liraglutide was obtained from Bachem (Bubendorf, Switzerland) and as a gift from Lotte Bjerre Knudsen at Novo Nordisk (Copenhagen, Denmark).

### Statistical analysis

Statistical analysis was performed using GraphPad Prism 6 software. Results were presented as mean ± standard error of the mean (SEM). The data with *P* < 0.05 were considered as significant. Paired Student’s t test was used for the paired data sets that were normally distributed. Wilcoxon matched-pairs signed rank test was used for the paired data sets that were not normally distributed. The outliers were detected by using Tukey method. Exclusion of the outlier was done before running the statistical analysis. One-way ANOVA with Bonferroni post hoc test was used for multiple comparisons.

## Results

The GABA-A-activated whole-cell currents were recorded in hippocampal CA3 pyramidal neurons in rat brain slices bathed in ACSF containing kynurenic acid. At the end of each experiment the GABA-A receptors specific antagonist bicuculline (100 μM) was added to inhibit the GABA-activated currents. We have previously shown that the CA3 pyramidal neurons have prominent synaptic and tonic currents that are transiently enhanced by GLP-1 and exendin-4 [19, 20]. Here we examined the effects of the GLP-1 analog liraglutide on these currents.

### Differential effects of liraglutide on GABA-activated synaptic transmission in CA3 pyramidal neurons

**Fig. 1:**
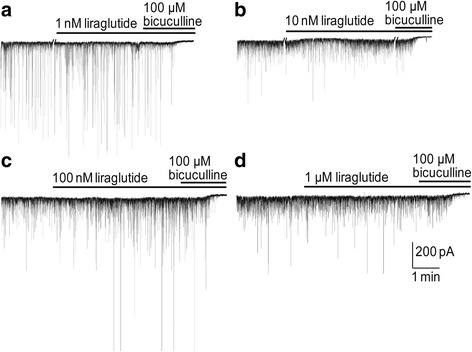
GABA-A receptor-mediated sIPSCs are enhanced by 100 nM liraglutide in hippocampal CA3 pyramidal neurons. Representative current traces show the effects of 1 nM (**a**), 10 nM (**b**), 100 nM (**c**) or 1 μM (**d**) liraglutide on the currents. Horizontal bars above the current recordings indicate the drug application periods. Sloped double lines in (**a**) and (**b**) indicate breaks in the recording

**Fig. 2:**
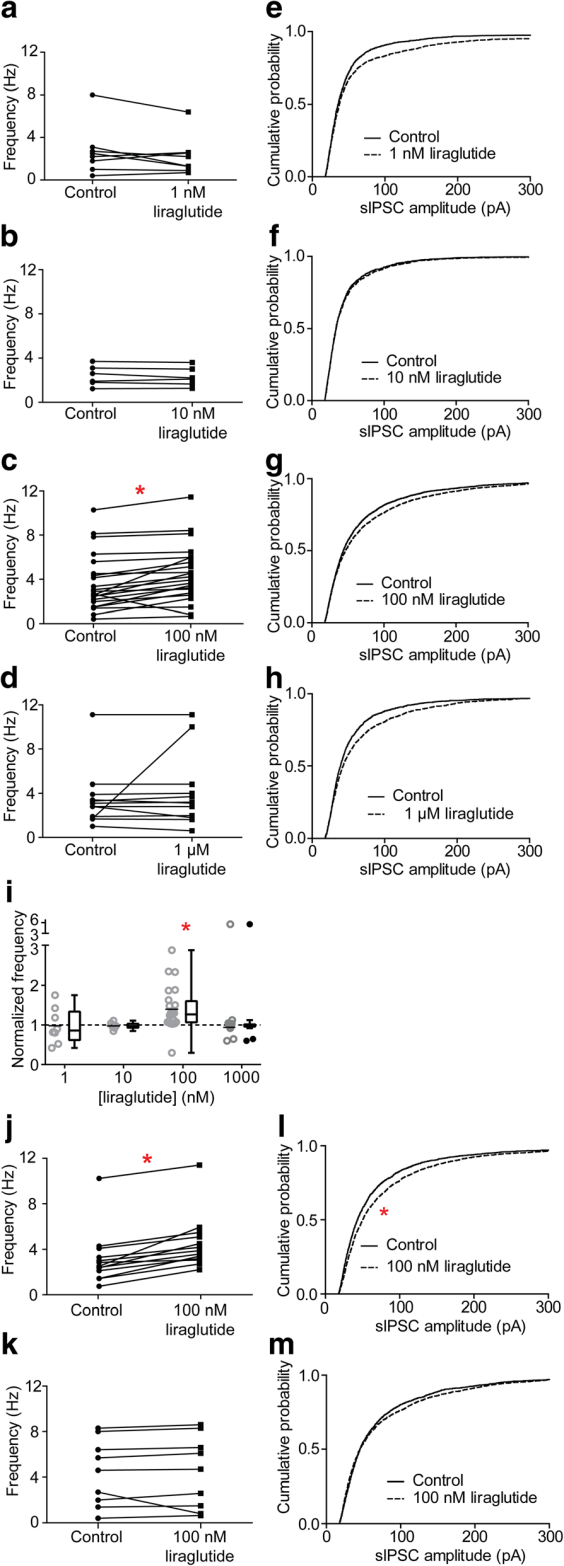
Liraglutide (100 nM) enhanced sIPSC frequency and amplitude in a subpopulation of CA3 pyramidal neurons. Liraglutide significantly enhanced the frequency of sIPSCs only at 100 nM (**c**, *n* = 23) but not 1 nM (**a**, *n* = 8), 10 nM (**b**, *n* = 6) or 1 μM (**d**, *n* = 12) (Wilcoxon matched-pairs signed rank test: *p < 0.05). Data are presented as • for control and ■ for drug application, and each connecting line indicates an individual cell. Cumulative probability histograms revealed no significant change of sIPSC amplitudes before (solid lines) and after (dash lines) the application of 1 nM (**e**, *n* = 8), 10 nM (**f**, *n* = 6), 100 nM (**g**, *n* = 23) and 1 μM (**h**, n = 12) liraglutide. (**i**) The normalized frequency of sIPSCs in control for each liraglutide concentration is shown by the horizontal dash line and the effects of the different concentration as a scatter dot plot (○) with a mean and a box-and-whiskers plot with median values plotted by Tukey method for detecting the outliers (•) above or below the box-and-whiskers plot. Statistical analyses were performed after outlier exclusion. One-way ANOVA Bonferroni post hoc test, multiple comparisons versus control group, *p < 0.05 for 100 nM liraglutide. Neurons in 100 nM liraglutide were grouped according to increase in frequency after liraglutide application, (**j**) > 20%, (**k**) < 20%, Wilcoxon matched-pairs signed rank test: *p < 0.05, *n* = 13. (**l**), (**m**) Cumulative probability histograms of sIPSC amplitudes of the corresponding subgroups of neurons from (**j**) and (**k**) exposed to100 nM liraglutide application. Solid and dash lines indicate cumulative probability histogram of sIPSCs before and after application of 100 nM liraglutide, respectively. Wilcoxon matched-pairs signed rank test: *p < 0.05, n = 13

We examined if liraglutide at concentrations ranging from 1 nM to 1 μM affected the GABA-evoked currents. Representative results are shown in Fig. 1. Liraglutide appeared to only enhance the synaptic GABA-activated currents at 100 nM. We examined further the effects on the spontaneous inhibitory postsynaptic currents (sIPSCs) and the results are shown in Fig. 2. The frequency responses, in ACSF alone (Control) and in liraglutide for the same neuron, are shown in Fig. 2a-d (a: 1 nM, b: 10 nM, c: 100 nM, d: 1 μM) and paired by the solid line for each neuron. Fig. 2e-h (e: 1 nM, f: 10 nM, g: 100 nM, h: 1 μM) shows the corresponding cumulative probability histograms of sIPSC amplitudes for the controls (solid line) and after liraglutide (broken line) had been applied to the neurons. Only in 100 nM liraglutide was there a prominent potentiation of the synaptic currents (Fig. 2c) resulting from an average increase in frequency of the sIPSCs (Fig. 2c and i). In contrast, there was no significant increase in the most probable current amplitude recorded at any liraglutide concentration (Fig. 2e-h). On closer inspection of the dataset where 100 nM liraglutide had been applied, it became apparent that in about 50% of the neurons the increase in frequency was more than 20% compared to the remaining neurons. We, therefore, regrouped these neurons and examined again if liraglutide modulated the frequency and amplitude of the synaptic currents. Fig. 2j-m shows that in neurons where liraglutide significantly increased the frequency of the synaptic currents by more than 20%, then the most probable synaptic current amplitudes were also significantly increased (Fig. 2j and l). This is in contrast to the second 100 nM liraglutide group where the frequency of the currents was increased slightly, less than 20% (Fig. 2k and m); here liraglutide had no significant effect on either the frequency or the amplitude of the synaptic currents. The results suggest that only a subpopulation of the neurons responds to liraglutide.

**Fig. 3:**
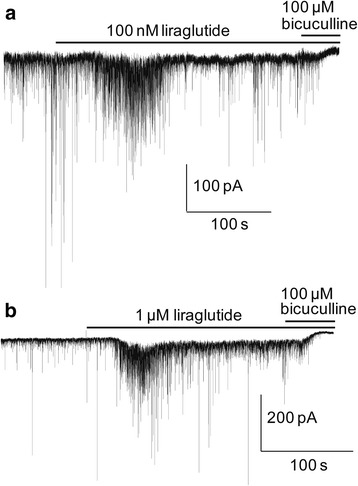
GABA-activated tonic currents. Currents recorded from two neurons where 100 nM (**a**) and 1 μM (**b**) liraglutide potentiated GABA-A receptor mediated tonic (and synaptic) currents with a peak tonic current amplitude of 20 pA and 88 pA, respectively. Horizontal bars above the current recordings indicate the drug application periods

We have previously shown that GLP-1 and exendin-4 induce a prominent transient enhancement of the tonic GABA-activated currents in hippocampal CA3 pyramidal neurons [19, 20]. Surprisingly, only in two of the 59 neurons in this study did we record effects of liraglutide on the tonic current (Fig. 3a and b) where the induced peak tonic current was 20 pA (100 nM liraglutide, Fig. 3a) and 88 pA (1 μM liraglutide, Fig. 3b).

### The synaptic currents are not enhanced by liraglutide in the presence of tetrodotoxin

**Fig. 4:**
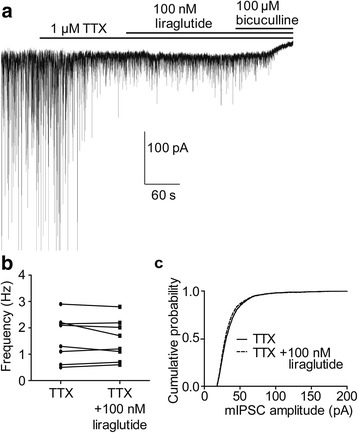
The mIPSCs are not enhanced by liraglutide when co-applied with TTX. (**a**) TTX (1 μM) decreased the frequency and amplitude of the IPSCs and the effect was maintained when 100 nM liraglutide was added. No significant increase of sIPSC frequency (**b**) or amplitude (**c**) between TTX (•) and TTX + 100 nM (■) liraglutide application (*n* = 8). Solid and dash lines in (**c)** indicate cumulative probability histogram of mIPSCs in the presence of TTX before and after application of liraglutide, respectively (*n* = 8)

In order to examine if the liraglutide effects on the currents were due to the pre- or postsynaptic mechanisms or both, we studied the influence of liraglutide on the currents in the presence of the voltage-gated sodium channel blocker tetrodotoxin (TTX, 1 μM). TTX inhibits action potential-dependent GABA release and decreases the frequency of GABA-activated synaptic currents to about 2 Hz in hippocampal CA3 pyramidal neurons [19]. Fig. 4a-c show that the frequency and most probable amplitude in TTX and TTX plus 100 nM liraglutide were similar and on the average 1.6 ± 0.3 Hz, 31 ± 2 pA and 1.5 ± 0.3 Hz, 30 ± 2 pA, respectively. The results are consistent with liraglutide potentiating GABA release from the presynaptic terminals.

## Discussion

In recent years a number of studies have highlighted the modulation by metabolic hormones of brain functions and the adverse effects that may arise if the signaling is malfunctioning [8, 13, 22–24]. The CA3 pyramidal neurons form an essential part of the hippocampal primary neurocircuitry. The hippocampus is the brain structure that is the center for memory and learning and, in addition, has a relatively unexplored role in metabolic functions [1, 3, 4]. We recently reported that GLP-1 and its mimetic exendin-4 transiently enhanced synaptic and tonic GABA-activated currents in these neurons by both pre- and postsynaptic mechanisms [19, 20]. We have now extended these studies and examined the effects of the GLP-1 analog, liraglutide. Our results reveal, somewhat surprisingly, that liraglutide only enhances the GABA-activated synaptic currents in a subset of the hippocampal CA3 pyramidal neurons and then, predominantly by presynaptic mechanisms.

**Fig. 5:**
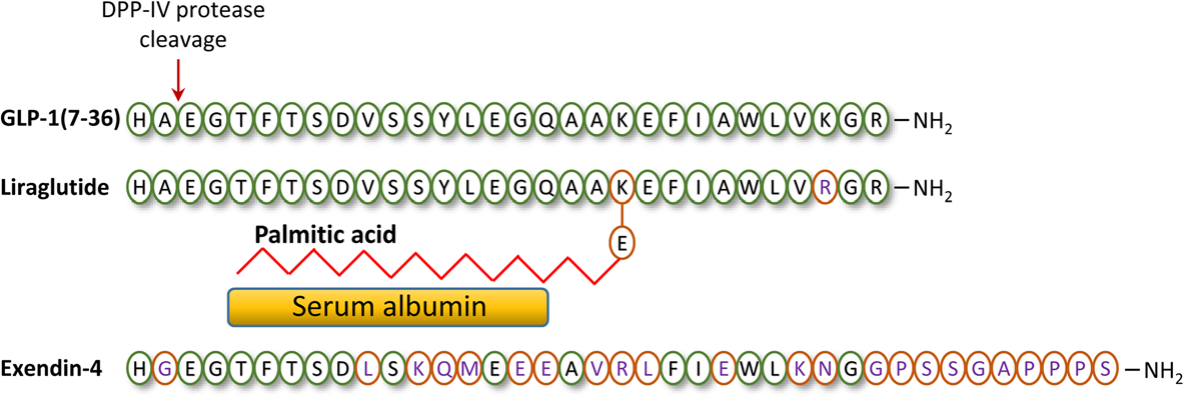
Amino acid sequences of GLP-1, liraglutide and exendin-4. Differences in amino acids are highlighted with orange ovals and violet letters. DPP-IV protease cleavage site is marked. C-16 fatty acid (palmitic acid) is linked to the peptide through glutamic acid spacer enabling binding to albumin and, thereby, preventing degradation by DPP-IV

It is commonly assumed that the GLP-1 receptor agonists, like GLP-1, exendin-4 and liraglutide, share the same basic signaling mechanisms [11, 13, 21] despite the knowledge that the GLP-1 receptor activation may result in more than one-type of signaling cascades [25–27]. Fig. 5 shows the amino acid sequence of the GLP-1, exendin-4 and liraglutide. Exendin-4 is a protein with 53% amino acid homology to the native human GLP-1 protein and was originally isolated from saliva of the lizard *Heloderma suspectum* [21]. Exendin-4 and its synthetic form exenatide activate the GLP-1 receptor with equal potency to GLP-1 [21]. Liraglutide, on the other hand, is a structural analog of GLP-1 with 97% amino acid homology to the native GLP-1 protein, with one amino acid substitution and a 16-carbon fatty acid side chain at the level of Lys26 [21]. Both exenatide and liraglutide in type-2 diabetic patients improve body weight and glycemic control without hypoglycemia [21] and, GLP-1 and exendin-4 similarly enhanced pre- and postsynaptic GABA signaling in hippocampal CA3 neurons [19]. The current study, however, demonstrates that liraglutide regulates the GABA signaling system in rat hippocampal CA3 pyramidal neurons somewhat differently from GLP-1 or exendin-4. It appears that concentrations lower than the 100 nM liraglutide used in our experiments are not high enough to trigger a response that can modulate the GABA signaling system whereas concentrations larger than 100 nM may e.g. desensitize the GLP-1 receptor, and thus, again no effect of liraglutide is recorded. Whether the difference is related to variable efficiency in activating the signaling cascades or if it depends on the level of activation of the GLP-1 receptor or the concentration level of the specific intracellular signaling molecules remains to be determined. It has indeed been shown that the GLP-1 receptor, which is a G-protein coupled receptor, activates the G-protein Gαs, which then activates adenyl cyclase resulting in an increase in the intracellular cAMP level [28, 29]. Notably, a few studies have also shown that GLP-1 can even activate Gα_i/O_ and Gα_q/1l_ [25, 26], at physiological GLP-1 concentrations in human and mouse pancreatic islets [27], resulting in activation of phospholipase C (PLC) and increased diacylglycerol and proteinkinase C (PKC) activity. It has been suggested that distinct domains within the third intracellular loop of the GLP-1 receptor are responsible for the activation of the different G-protein subfamilies [25, 26]. It is clearly feasible that the various agonists at the GLP-1 receptor will vary in their efficacy of inducing the appropriate activation of the diverse G-proteins and the respective intracellular signaling cascades in the neurons.

## Conclusion

In summary, the GLP-1 receptor agonists liraglutide, exendin-4 and GLP-1 differentially regulate GABA-activated signaling in rat hippocampal CA3 pyramidal neurons. The results are consistent with variable efficacy of agonists at the GLP-1 receptor.

## Abbreviations

ACSF: Artificial cerebrospinal fluid
GABA: γ-aminobutyric acid
GLP-1: Glucagon-like peptide-1
s/mIPSC: Spontaneous/miniature inhibitory postsynaptic current
TTX: Tetrodotoxin

## References

[1] Strange BA, Witter MP, Lein ES, Moser EI (2014). Functional organization of the hippocampal longitudinal axis. Nat Rev Neurosci.

[2] Zeidman P, Maguire EA (2016). Anterior hippocampus: the anatomy of perception, imagination and episodic memory. Nat Rev Neurosci.

[3] Lathe R (2001). Hormones and the hippocampus. J Endocrinol.

[4] Risold PY, Swanson LW (1996). Structural evidence for functional domains in the rat hippocampus. Science.

[5] Reimann F, Ward PS, Gribble FM (2006). Signaling mechanisms underlying the release of glucagon-like peptide 1. Diabetes.

[6] Lim GE, Brubaker PL (2006). Glucagon-like peptide 1 secretion by the L-cell - the view from within. Diabetes.

[7] Moran C, Beare R, Phan TG, Bruce DG, Callisaya ML, Srikanth V (2015). Neuroimaging AsD: type 2 diabetes mellitus and biomarkers of neurodegeneration. Neurology.

[8] Willette AA, Modanlo N, Kapogiannis D (2015). Neuroimaging AsD: insulin resistance predicts medial temporal Hypermetabolism in mild cognitive impairment conversion to Alzheimer disease. Diabetes.

[9] Sebastiao I, Candeias E, Santos MS, de Oliveira CR, Moreira PI, Duarte AI (2014). Insulin as a bridge between type 2 diabetes and Alzheimer disease - how anti-diabetics could be a solution for dementia. Front Endocrinol (Lausanne).

[10] Claxton A, Baker LD, Hanson A, Trittschuh EH, Cholerton B, Morgan A, Callaghan M, Arbuckle M, Behl C, Craft S (2015). Long-acting intranasal insulin Detemir improves cognition for adults with mild cognitive impairment or early-stage Alzheimer's disease dementia (vol 44, pg 897, 2015). Journal of Alzheimers Disease.

[11] Yarchoan M, Arnold SE (2014). Repurposing diabetes drugs for brain insulin resistance in Alzheimer disease. Diabetes.

[12] Cork SC, Richards JE, Holt MK, Gribble FM, Reimann F, Trapp S (2015). Distribution and characterisation of glucagon-like peptide-1 receptor expressing cells in the mouse brain. Mol Metab.

[13] Holst JJ, Burcelin R, Nathanson E (2011). Neuroprotective properties of GLP-1: theoretical and practical applications. Curr Med Res Opin.

[14] Olsen RW, Sieghart W (2008). International Union of Pharmacology. LXX. Subtypes of gamma-aminobutyric acid(a) receptors: classification on the basis of subunit composition, pharmacology, and function. Update. Pharmacol Rev.

[15] Semyanov A, Walker MC, Kullmann DM, Silver RA (2004). Tonically active GABA a receptors: modulating gain and maintaining the tone. Trends Neurosci.

[16] Palovcik RA, Phillips MI, Kappy MS, Raizada MK (1984). Insulin inhibits pyramidal neurons in hippocampal slices. Brain Res.

[17] Wan Q, Xiong ZG, Man HY, Ackerley CA, Braunton J, WY L, Becker LE, MacDonald JF, Wang YT (1997). Recruitment of functional GABA(a) receptors to postsynaptic domains by insulin. Nature.

[18] Jin Z, Jin Y, Kumar-Mendu S, Degerman E, Groop L, Birnir B (2011). Insulin reduces neuronal excitability by turning on GABA(a) channels that generate tonic current. PLoS One.

[19] Korol SV, Jin Z, Babateen O, Birnir B (2015). Glucagon-like peptide-1 (GLP-1) and exendin-4 transiently enhance GABAA receptor-mediated synaptic and tonic currents in rat hippocampal CA3 pyramidal neurons. Diabetes.

[20] Korol SV, Jin Z, Birnir B. The GLP-1 receptor agonist Exendin-4 and diazepam differentially regulate GABA(a) receptor-mediated tonic currents in rat hippocampal CA3 pyramidal neurons. PLoS One. 2015;10(4) 10.1371/journal.pone.0124765 PMC4415774 25927918

[21] Lund A, Knop FK, Vilsboll T (2014). Glucagon-like peptide-1 receptor agonists for the treatment of type 2 diabetes: differences and similarities. Eur J Intern Med.

[22] Biessels GJ, Strachan MW, Visseren FL, Kappelle LJ, Whitmer RA (2014). Dementia and cognitive decline in type 2 diabetes and prediabetic stages: towards targeted interventions. Lancet Diabetes Endocrinol.

[23] Cui Y, Jiao Y, Chen YC, Wang K, Gao B, Wen S, SH J, Teng GJ (2014). Altered spontaneous brain activity in type 2 diabetes: a resting-state functional MRI study. Diabetes.

[24] Secher A, Jelsing J, Baquero AF, Hecksher-Sorensen J, Cowley MA, Dalboge LS, Hansen G, Grove KL, Pyke C, Raun K (2014). The arcuate nucleus mediates GLP-1 receptor agonist liraglutide-dependent weight loss. J Clin Invest.

[25] Bavec A, Hallbrink M, Langel U, Zorko M (2003). Different role of intracellular loops of glucagon-like peptide-1 receptor in G-protein coupling. *Regul*. Peptides.

[26] Hallbrink M, Holmqvist T, Olsson M, Ostenson CG, Efendic S, Langel U (2001). Different domains in the third intracellular loop of the GLP-1 receptor are responsible for G alpha(s) and G alpha(i)/G alpha(o) activation. Bba-Protein Struct M.

[27] Shigeto M, Ramracheya R, Tarasov AI, Cha CY, Chibalina MV, Hastoy B, Philippaert K, Reinbothe T, Rorsman N, Salehi A (2015). GLP-1 stimulates insulin secretion by PKC-dependent TRPM4 and TRPM5 activation. J Clin Invest.

[28] Roed SN, Wismann P, Underwood CR, Kulahin N, Iversen H, Cappelen KA, Schaffer L, Lehtonen J, Hecksher-Soerensen J, Secher A (2014). Real-time trafficking and signaling of the glucagon-like peptide-1 receptor. Mol Cell Endocrinol.

[29] Holst JJ (2007). The physiology of glucagon-like peptide 1. Physiol Rev.

